# Monoterpenoid Terpinen-4-ol Exhibits Anticonvulsant Activity in Behavioural and Electrophysiological Studies

**DOI:** 10.1155/2014/703848

**Published:** 2014-08-10

**Authors:** Franklin F. F. Nóbrega, Mirian G. S. S. Salvadori, Cintia J. Masson, Carlos F. Mello, Tiago S. Nascimento, José H. Leal-Cardoso, Damião P. de Sousa, Reinaldo N. Almeida

**Affiliations:** ^1^Programa de Pós-Graduação em Biotecnologia, Rede Nordeste de Biotecnologia (RENORBIO), Caixa Postal 5009, 58051-900 João Pessoa, PB, Brazil; ^2^Unidade Acadêmica de Tecnologia do Desenvolvimento, Centro de Desenvolvimento Sustentável do Semiárido, Universidade Federal de Campina Grande, 58540-000 Sumé, PB, Brazil; ^3^Laboratório de Psicofarmacologia, Centro de Ciências da Saúde, Universidade Federal da Paraíba, Caixa Postal 5009, 58051-900 João Pessoa, PB, Brazil; ^4^Departamento de Fisiologia e Farmacologia, Universidade Federal de Santa Maria, 97105-900 Santa Maria, RS, Brazil; ^5^Laboratório de Eletrofisiologia, Instituto Superior de Ciências Biomédicas, Universidade Estadual do Ceará, Campus do Itaperi, 60740-000 Fortaleza, CE, Brazil; ^6^Departamento de Ciências Farmacêuticas, Centro de Ciências da Saúde, Universidade Federal da Paraíba, 58051-900 João Pessoa, PB, Brazil; ^7^Departamento de Fisiologia e Patologia, Centro de Ciências da Saúde, Universidade Federal da Paraíba, 58051-900 João Pessoa, PB, Brazil

## Abstract

Terpinen-4-ol (4TRP) is a monoterpenoid alcoholic component of essential oils obtained from several aromatic plants. We investigated the psychopharmacological and electrophysiological activities of 4TRP in male Swiss mice and Wistar rats. 4TRP was administered intraperitoneally (i.p.) at doses of 25 to 200 mg/kg and intracerebroventricularly (i.c.v.) at concentrations of 10, 20, and 40 ng/2 *μ*L. For in vitro experiments, 4TRP concentrations were 0.1 mM and 1.0 mM. 4TRP (i.p.) inhibited pentylenetetrazol- (PTZ-) induced seizures, indicating anticonvulsant effects. Electroencephalographic recordings showed that 4TRP (i.c.v.) protected against PTZ-induced seizures, corroborating the behavioural results. To determine whether 4TRP exerts anticonvulsant effects via regulation of GABAergic neurotransmission, we measured convulsions induced by 3-mercapto-propionic acid (3-MP). The obtained results showed involvement of the GABAergic system in the anticonvulsant action exerted by 4TRP, but flumazenil, a selective antagonist of the benzodiazepine site of the GABA_A_ receptor, did not reverse the anticonvulsant effect, demonstrating that 4TRP does not bind to the benzodiazepine-binding site. Furthermore, 4TRP decreased the sodium current through voltage-dependent sodium channels, and thus its anticonvulsant effect may be related to changes in neuronal excitability because of modulation of these channels.

## 1. Introduction

Natural products have been recognized as productive sources from which compounds of pharmacological interest are obtained [1]. Empirical investigation by the scientific research community to determine the mechanisms through which such compounds produce their effects will allow for their use in a manner free of adverse effects [2].

Because of their multiple pharmacological actions, there are many reports of the use of medicinal plants and their constituents for the treatment of human diseases. For example, aromatic plants have often been used in such a manner. Important actions in the central nervous system (CNS) produced by these plants, such as anxiolytic [3], analgesic [4], sedative, and anticonvulsant activity [5], are generally attributed to the essential oils.

Epilepsy is a chronic neurological disease characterized by spontaneous recurrent seizures that affects approximately 1% of the world population. Nearly 30% of patients with epilepsy do not respond to any form of pharmacological treatment [6]. For these patients, currently available antiepileptic drugs are ineffective and have significant undesirable effects. For these reasons, numerous studies have been aimed at the discovery of new pharmacological strategies using novel compounds to treat epilepsy.

Terpenes are a diverse class of organic compounds frequently found in high concentrations in volatile oils from plants. Monoterpenes exhibit diverse pharmacological effects, including antinociceptive [7, 8] and anticonvulsant [9] activity. The ability of these substances to inhibit or abolish seizures merits research attention.

The monoterpenoid terpinen-4-ol (4TRP) is a major component of several species of aromatic plants [10]. Plants that produce this substance have been shown to have important biological activities such as cardiovascular [11] and antioxidant [12] effects.

**Figure 1:**
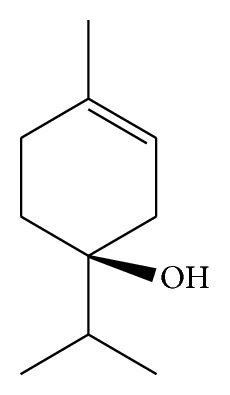
Chemical structure of terpinen-4-ol (4TRP).

In previous studies, 4TRP (Figure 1) produced a depressant effect on the CNS and also exhibited a significant anticonvulsant effect in animal seizure models, including models in which convulsions are induced by electroshock and picrotoxin [13].

Therefore, the present study investigated the effects of 4TRP on convulsions induced by mercaptopropionic acid (3-MP) and seizures induced by pentylenetetrazol (PTZ) via behavioural and electroencephalographic methods and investigated the molecular mechanisms involved in these effects.

## 2. Material and Methods

### 2.1. Animals and Reagents

Adult male Swiss mice (25–30 g) and Wistar rats (200–300 g) were used. The animals were obtained from the Dr. George Thomas Bioterium, Federal University of Paraíba, and randomly housed in rodent cages in a controlled environment (12 h light/dark cycle, 24 ± 1°C, 55% relative humidity) with free access to food and water. Animals were used in groups of 8. All animals were acclimatized before the experiments, and the behavioural observations were conducted between 08:00 and 13:00 h. Experimental protocols and procedures were approved by the local animal ethics committee (CEPA/LTF-UFPB 0202/08; CEUA-UECE: 10339956-9). All reagents were purchased from Sigma-Aldrich (St. Louis, MO, USA).

### 2.2. PTZ-Induced Convulsions

The evaluation of anticonvulsant activity was carried out by using the PTZ test [14, 15]. This test was chosen because it has a good predictive value for anticonvulsant and antiepileptic activities in the clinic [16, 17]. Mice were divided into 6 groups (*n* = 8 per group). The negative and positive control groups received 5% Tween 80 or 4 mg/kg diazepam (DZP), respectively. The remaining groups were intraperitoneally (i.p.) injected with 4TRP (25, 50, 75, or 150 mg/kg). Thirty minutes after drug administration, the mice were injected with PTZ (60 mg/kg, i.p.) and observed for at least 15 min to detect the occurrence of the first episode of forelimb clonus.

### 2.3. Mercaptopropionic Acid (3-MP) Test

3-MP is a relatively specific inhibitor of glutamate decarboxylase, which catalyses the formation of *γ*-aminobutyric acid (GABA), the major inhibitory neurotransmitter of the CNS. The inhibition of seizures induced by 3-MP acid is a well-established method with which drugs with effects on the GABAergic system are evaluated [18]. Mice were divided into 5 groups (*n* = 8 in each group). The negative and positive control groups received 5% Tween 80 or 4 mg/kg DZP, respectively. The remaining groups received 4TRP (50, 100, or 200 mg/kg, i.p.). Thirty minutes after drug administration, the mice received 3-MP (70 mg/kg, i.p.) and were observed for at least 25 min to detect the occurrence of the first episode of forelimb clonus.

### 2.4. Evaluation of the Participation of the Benzodiazepine Site of the GABA_A_ Receptor

3-MP is a relatively specific inhibitor of glutamate decarboxylase, which catalyses the formation of *γ*-aminobutyric acid (GABA), the major inhibitory neurotransmitter of the CNS. The inhibition of seizures induced by 3-MP acid is a well-established method with which drugs with effects on the GABAergic system are evaluated [18]. Mice were divided into 5 groups (*n* = 8 in each group). The negative and positive control groups received 5% Tween 80 or 4 mg/kg DZP, respectively. The remaining groups received 4TRP (50, 100, or 200 mg/kg, i.p.). Thirty minutes after drug administration, the mice received 3-MP (70 mg/kg, i.p.) and were observed for at least 25 min to detect the occurrence of the first episode of forelimb clonus.

### 2.5. PTZ Test: EEG Recordings

EEG recordings and intracerebral injections of drugs were performed as described by Cavalheiro [19]. Using stereotaxic guidance, cannulae and electrodes were implanted in specific brain regions. Animals were anaesthetized with ketamine (100 mg/kg, i.p.) and xylazine (30 mg/kg, i.p.) and placed in a stereotaxic apparatus. A cannula was inserted unilaterally in the right lateral ventricle (coordinates relative to the bregma: AP 4.5 mm and L 2.5 mm from the dura) [20]. Two stainless steel screw electrodes were placed bilaterally on the parietal cortex, along with a ground lead positioned over the nasal sinus. The electrodes were connected to a multipin socket and, together with the injection cannula, were fixed to the skull with dental acrylic cement. All experiments were performed 5 days after surgery.

4TRP was intracerebroventricularly (i.c.v.) administered using a needle (30-gauge) protruding 1 mm below the cannula guide, which was glued to a multipin socket and inserted through a previously opened skull orifice. All doses of 4TRP (10, 20, or 40 ng/2 *μ*L, i.c.v.) or vehicle (Tween 80) were injected over a period of 1 min using a Hamilton syringe (Hamilton Co., Reno, NV, USA), and an additional minute was allowed to elapse before removal of the needle to avoid backflow of drug through the cannula. Thirty minutes after the administration, seizures were induced by the administration of PTZ (60 mg/kg, i.p.). After PTZ administration, animals were transferred to an open field apparatus (54.7 cm diameter), where they were monitored by a video camera for a period of 20 min. During this time, clonic seizures and generalized convulsions were recorded.

The subject was then connected to the lead socket on a swivel inside of a Faraday cage. To establish an adequate control period, EEG signals were recorded for 10 minutes using a digital encephalograph (Neuromap EQSA260, Neuromap LTDA, Brazil). EEG signals were amplified, filtered (0.1–70.0 Hz, bandpass), digitized (256 Hz sampling rate), and stored in a PC for offline analysis. EEG recordings were analysed visually to verify the occurrence of events that could lead to artefacts [21]; such recordings were easily identified and discarded.

### 2.6. Dissociation and Patch-Clamp Recordings

The dorsal root ganglion (DRG) was collected from the rats and placed in Ca^2+^- and Mg^2+^-free Hanks' balanced salt solutions. Next DRG was placed in dissociation solutions: 1 mg/mL collagenase type I for 65 min and 2.5 mg/mL trypsin with 0.25 mg/mL EDTA for 15 min, both in Hanks' balanced salt solution at 37°C. After exposure to the dissociation solutions, the DRG neurons were freed from the tissue by gentle trituration in Dulbecco's modified Eagle's medium containing 10% foetal bovine serum, 100 U/mL streptomycin, and 0.1 mg/mL penicillin. The cells were plated on coverslips coated with 0.01% poly-D-lysine. The neurons were incubated in an atmosphere of air containing 5% CO_2_ maintained at 37°C and were used within 48 h.

The whole-cell patch-clamp recordings were performed in a voltage-clamp configuration as described by Leal-Cardoso et al. [22]. Briefly, coverslips with dissociated DRG neurons were placed in a chamber on an inverted phase contrast microscope and maintained in a bath solution until the recording was begun. A perfusion pipette was positioned near the cell to be challenged. Thick-walled flint glass tubing (1.5 mm outside diameter, 1.1 mm inside diameter; Perfecta, São Paulo, Brazil) was pulled with a Flaming/Brown type puller (P-97 micropipette puller, Sutter Instruments, Novato, CA, USA) to make the patch pipettes. Patch pipettes were filled with internal solution and had resistance ranging from 1.5 to 3.0 MΩ. Recordings were made using an Axopatch 200B amplifier driven by Clampex software (Molecular Devices, Sunnyvale, CA, USA). The holding potential was set at −80 mV for all experimental manipulations. A 100 ms voltage step to 0 mV at 5 s intervals was employed to elicit the total Na^+^ current. The experimental time points were control with test solution perfusion only (approximately 30 s), drug exposure with test solution containing 4TRP (0.1 or 1.0 mM), washout/recovery, and further test solution perfusion. Capacitance and leakage subtraction were performed using a P/6 subtraction protocol. The current was sampled at 40 kHz and low-pass filtered at 5 kHz, and data acquisition and storage were performed using a computer acquisition hardware (Digidata 1440A, Molecular Devices, Sunnyvale, CA, USA).

### 2.7. Statistical Analysis

Data are shown as mean ± SEM or median and interquartile ranges. The results were analysed statistically by one-way analysis of variance (ANOVA), followed by Dunnett's test for normally distributed data (parametric tests) or the Kruskal-Wallis test followed by Dunn's test for nonparametric data. The Fischer exact test was used to perform analyses between 2 groups. A *P* value of *P* < 0.05 was considered to be significant, and *P* and *H* values are reported only if *P* < 0.05.

## 3. Results

### 3.1. Effect of 4-TRP on PTZ-Induced Seizures

**Figure 2:**
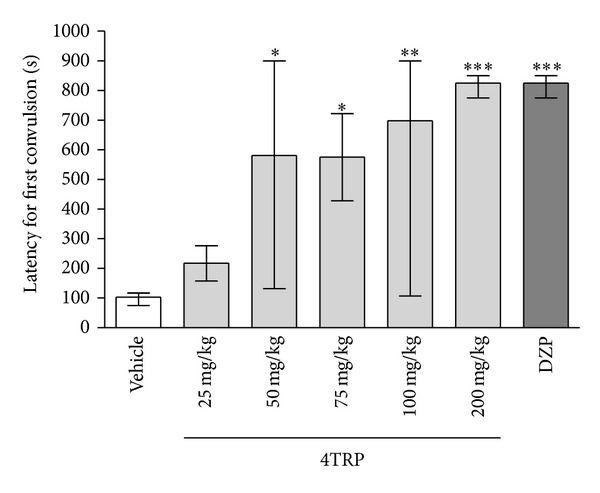
Effect of 4TRP on the latency to the development of PTZ-induced convulsions in mice. The values represent the median and interquartile ranges. One-way ANOVA/Dunn's test, ∗*P* < 0.05, ∗∗*P* < 0.01, ∗∗∗*P* < 0.001 compared with control values.

**Figure 3:**
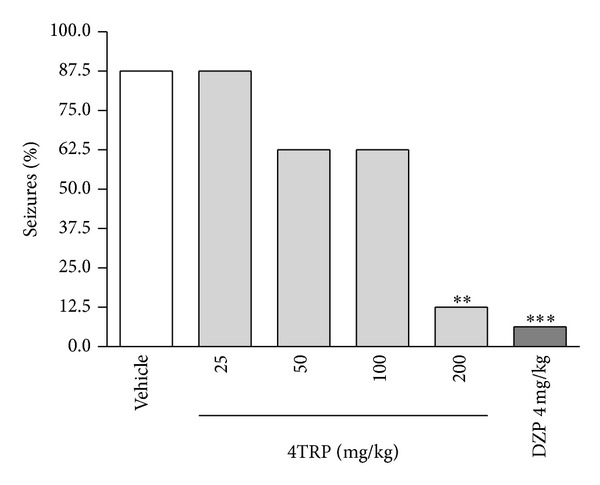
Protective effect of 4TRP against PTZ-induced seizures. The values represent the percentage of seizures in each group. Fisher's exact test, ∗∗*P* < 0.01, ∗∗∗*P* < 0.001 compared with control values.

Figure 2 shows the effects of increasing doses of 4TRP against PTZ-induced seizures. 4TRP at doses of 50 [581.0, (132.0–900.0)], 75 [616.0, (208.5–900.0)], and 150 mg/kg [825.0, (775.0–850.0)] significantly increased the latency to PTZ-induced seizures [*H*(3) = 14.67; *P* < 0.01] compared with the control group [100.0, (70.0–113.0)] (Kruskal-Wallis test followed by Dunn's test). Diazepam (4 mg/kg, i.p.) also decreased PTZ-induced seizures [825.0, (775.0–850.0)]. The seizure latency of the group injected with 4TRP (150 mg/kg) was not different from that of the group that received DZP.  Figure 3 shows the significant decrease in the number of animals that showed convulsive behaviour (4TRP, 200 mg/kg).

### 3.2. Effect of 4-TRP on 3-MP-Induced Convulsions

**Figure 4:**
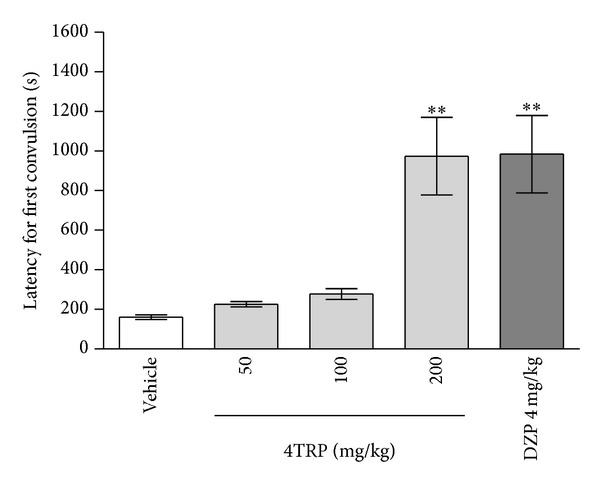
Effect of 4TRP on 3-mercaptopropionic acid- (3-MP-) induced seizures in mice. The latency to the onset of seizures was measured. Values represent mean ± S.E.M. (*n* = 8 per group). One-way ANOVA followed by Dunnett's test; ∗∗*P* < 0.01 compared to vehicle-treated mice (control).

Figure 4 shows the dose-related inhibition of 3-MP-induced seizures by systemic administration of 4TRP. Compared to vehicle (160.6 ± 12.3 s), 4TRP (200 mg/kg) significantly increased seizure latency (1064.0 ± 206.0 s, *P* < 0.01). DZP at a dose of 4 mg/kg (984.1 ± 195.3 s, *P* < 0.01) was used as the reference drug (positive control group) and produced a latency to onset of seizures that was similar to that of the experimental group (200 mg/kg 4TRP).

### 3.3. Evaluation of the Participation of the Benzodiazepine Site of the GABA_A_ Receptor

**Figure 5:**
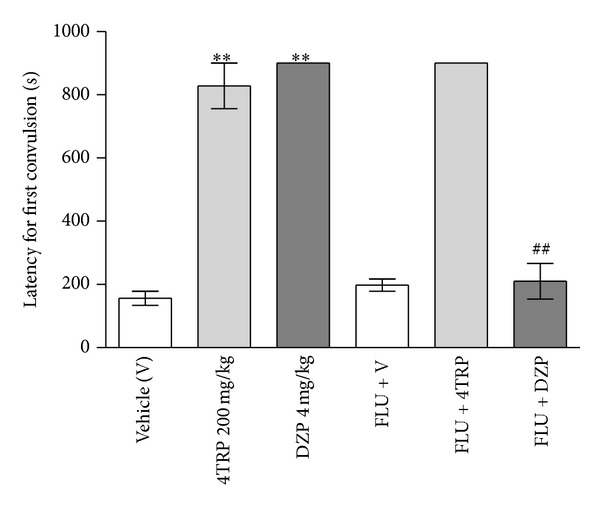
Effect of blockage of the benzodiazepine binding site (GABA_A_) by flumazenil on the effect of 4TRP on PTZ-induced seizures in mice. The values represent mean ± S.E.M. (*n* = 8 per group). One-way ANOVA followed by Dunnett's test, ∗∗*P* < 0.01 compared to vehicle-treated mice (control). Student's *t*-test, ^##^ *P* < 0.01 versus diazepam.

DZP (4 mg/kg, i.p.) produced a significant increase in seizure latency (900.0 ± 0.0 s, *P* < 0.01) as compared to the control group (156.0 ± 22.4 s) (Figure 5). This effect of DZP was abolished by pretreatment with FLU (20 mg/kg, s.c.) (210.1 ± 56.0 s). 4TRP at a dose of 200 mg/kg significantly increased the latency to the onset of convulsions (828.0 ± 72.0 s, *P* < 0.01) and protected the subjects from the development of seizures. However, this effect was not reversed by pretreatment with FLU (20 mg/kg, s.c.) (900.0 ± 0.0 s, *P* < 0.01).

### 3.4. Effect of TRP-4 on PTZ-Induced EEG Alterations

**Figure 6:**
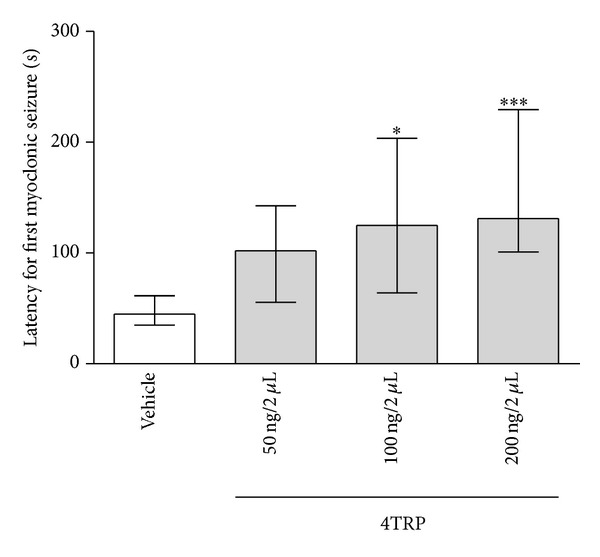
Effect of 4TRP on the latency to the development of the first myoclonic convulsion after PTZ treatment in mice. The values represent the median and interquartile ranges. One-way ANOVA/Dunn's test, ∗*P* < 0.05, ∗∗∗*P* < 0.001, as compared with control values.

**Figure 7:**
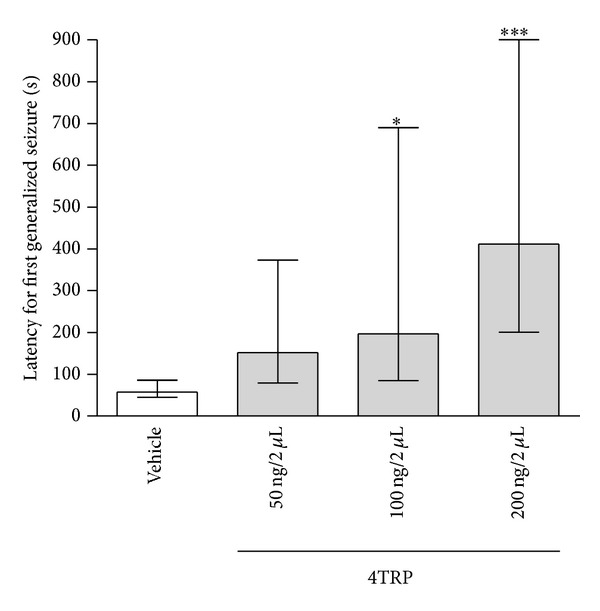
Effect of 4TRP on the latency to the development of the first generalized convulsion after PTZ treatment in mice. The values represent the median and interquartile ranges. One-way ANOVA/Dunn's test, ∗*P* < 0.05, ∗∗∗*P* < 0.001, as compared with control values.

Figure 6 shows the effect of 4TRP on the latency to the first myoclonic manifestation with EEG alteration after PTZ treatment. The latency to myoclonic manifestation with EEG alteration was increased in the groups administered 4TRP at a dose of 20 ng/2 *μ*L i.c.v. [125.0, (64.0–203.5); *H*(3) = 14.93; *P* < 0.05] or 40 ng/2 *μ*L i.c.v. [131.0, (101.0–229.5); *H*(1) = 14.93; *P* < 0.001] compared with the vehicle group [45.0, (35.0–61.5)]. The group injected with 4TRP at a dose of 20 ng/2 *μ*L i.c.v. [196.5, (84.5–690.0); *H*(3) = 17.94; *P* < 0.05] or 40 ng/2 *μ*L i.c.v. [411.5, (200.5–900.0); *H*(3) = 17.95; *P* < 0.001] showed increased latency to generalized tonic-clonic seizures as compared to the control group [57.0, (45.0–86.0)] (Figure 7).

**Figure 8:**
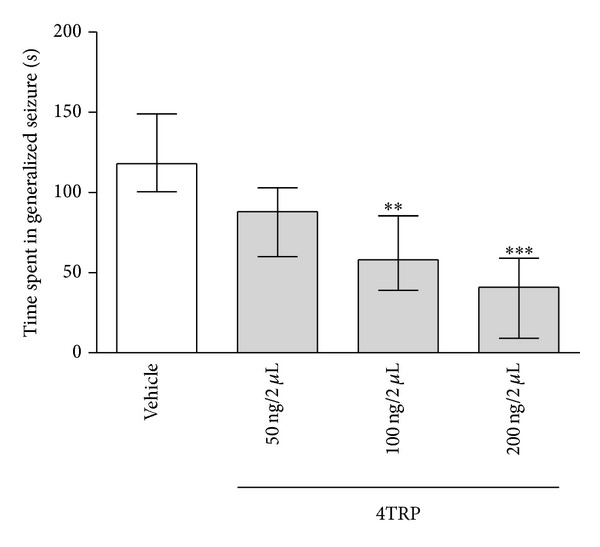
Effect of 4TRP on the time spent in generalized convulsions after PTZ treatment in mice. The values represent the median and interquartile ranges. One-way ANOVA/Dunn's test, ∗∗*P* < 0.01, ∗∗∗*P* < 0.001, as compared with control values.

4TRP also reduced the total time spent in generalized convulsions at doses of 20 ng/2 *μ*L [58.0, (39.0–85.5); *H*(3) = 24.32; *P* < 0.01] and 40 ng/2 *μ*L [41.0, (9.0–59.0); *H*(3) = 24.32; *P* < 0.001] (Figure 8). Statistical analysis (Dunn's multiple comparison nonparametric test) showed that 4TRP at doses 20 ng/2 *μ*L and 40 ng/2 *μ*L significantly reduced seizures.

**Figure 9:**
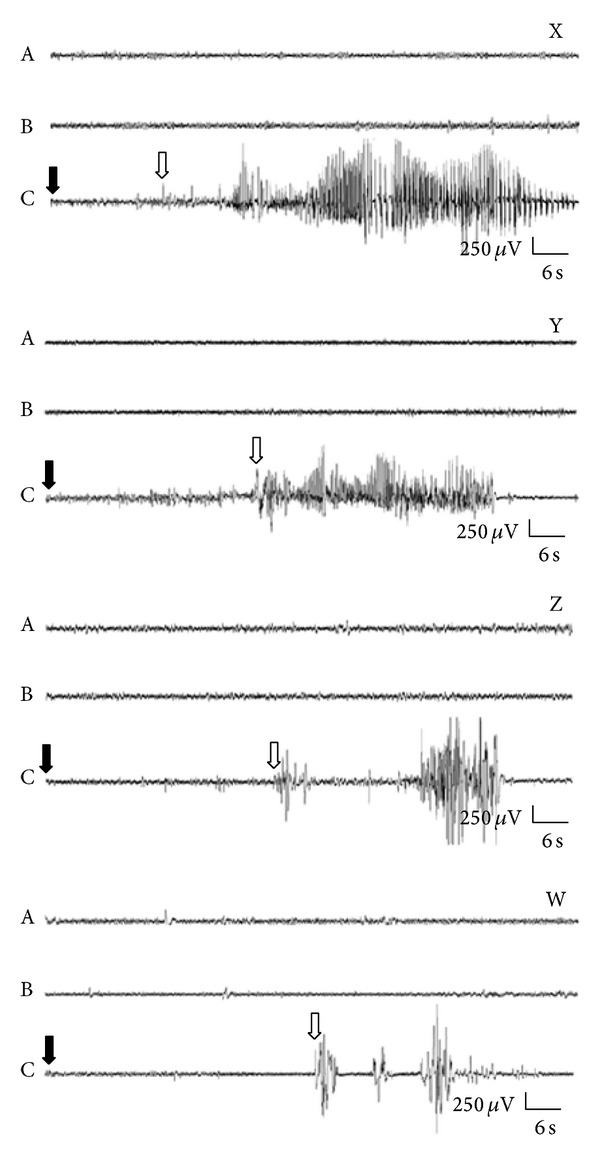
Representative electroencephalographic recordings from the parietal cortex (CTX) of animals during (A) basal activity or after administration of (B) vehicle or (C) PTZ (60 mg/kg, i.p.). Black arrows indicate the time of PTZ administration and white arrows indicate the onset of seizures. Recordings are shown for mice not administered 4TRP (X-C), as well as for those administered 4TRP at 10 ng/2 *μ*L (Y-C), 20 ng/2 *μ*L (Z-C), or 40 ng/2 *μ*L (B) (W-C).

The quantitative results reported above were corroborated by analysing the representative EEG records (Figure 9) after the administration of 4TRP at different concentrations. The time spent in generalized convulsion in the group that received 10 ng/2 *μ*L 4TRP (Figure 9(Y)) was higher than that in the groups that received 20 ng/2 *μ*L or 40 ng/2 *μ*L (Figures 9(Z) and 9(W)) but lower than that in the control group (Figure 9(X)), as indicated by the relative mean amplitudes of the EEG recordings.

### 3.5. Effect on Dissociated DRG Neurons

**Figure 10:**
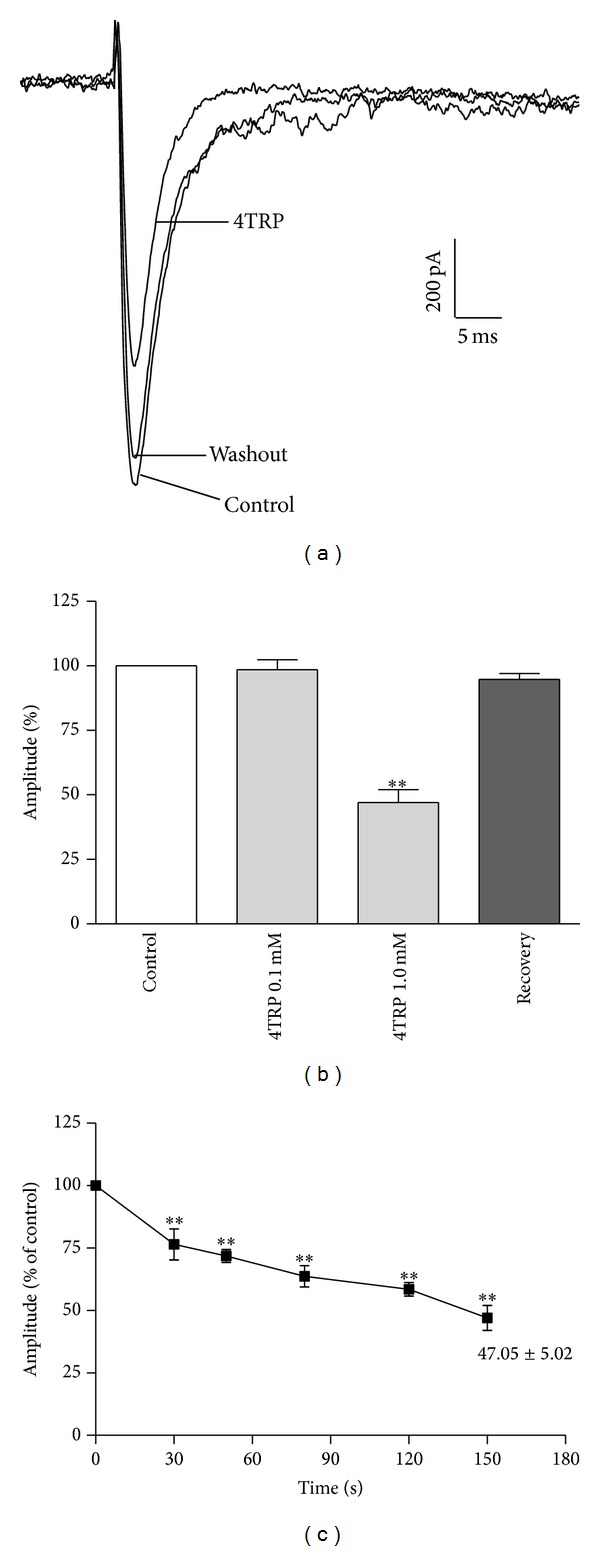
(a) Representative traces of Na^+^ current in the absence of 4TRP (control), in presence of 4TRP (1.0 mM), and after washing. (b) Amplitude of Na^+^ current in response to exposure to 4TRP at 0.1 mM and 1.0 mM. (c) The decay of the amplitude of the Na^+^ current in response to exposure to 1.0 mM 4TRP as a function of time (s). The values presented in (b) and (c) represent mean ± SEM (*n* = 5). One-way ANOVA followed by Dunnett's test, ∗∗*P* < 0.01 compared with the control (before exposure to 4TRP).

4TRP reduced Na^+^ currents in a concentration-dependent manner.  Figure 10(a) shows representative traces of Na^+^ currents in the absence of 4TRP (control), in presence of 4TRP (1.0 mM), and after washing. At a concentration of 1.0 mM, 4TRP significantly inhibited Na^+^ current (0.9 ± 2.78 nA; *P* < 0.01) compared with control treatment (5.68 ± 0.6 nA), reducing the peak amplitude of sodium to 47.1 ± 5.0% of the control value (100.0 ± 0.0). At a concentration of 0.1 mM, 4TRP did not significantly inhibit Na^+^ current (5.0 ± 1.1 nA) compared with the control treatment (4.92 ± 0.8 nA), which involved treatment with the vehicle only (0.2% dimethyl sulfoxide). The reduction in the peak amplitude of the Na^+^ current (Figure 10(b)) relative to the control cells occurred within approximately 150 s (Figure 10(c)), and this effect was reversible after washing (Figure 10(b)).

## 4. Discussion

The effects of the monoterpene 4TRP on the CNS have been reported previously. In behavioural experiments, 4TRP increased the sleep time induced by barbiturates, decreased locomotor activity at high doses, and significantly inhibited seizures [13].

Based on these results, which indicate that this substance has a potent inhibitory effect on the CNS, we prioritized the investigation of the anticonvulsant activity of this substance.

In this study, we investigated the anticonvulsant effects of i.c.v. and i.p. administered 4TRP by analysing EEG recordings and the convulsive behaviour induced by PTZ, which is a potent convulsant that inhibits chloride ion channels activated by *γ*-aminobutyric acid (GABA_A_ receptors). The inhibition of convulsions induced by PTZ is considered to be a predictive experimental model for convulsive crises of the generalized or clonic type [23]. In addition, compounds that show anticonvulsant activity in epilepsy models of partial seizures effectively inhibit convulsions in this model [24].

Results from the present study show that 4TRP effectively inhibited the incidence and severity of PTZ-induced seizures, as demonstrated by prolongation of latency to the initiation of convulsions at 4TRP doses lower than those reported previously [13]. This result is important because it verifies the presence of the anticonvulsive activity of 4TRP at doses that may produce fewer toxic effects, and it thus suggests a more favourable therapeutic index for 4TRP than was previously reported. These data are in agreement with the results obtained in mice administered *α*-terpineol, an analogue of terpinen-4-ol [25].

We investigated brain activity by analysing EEG records. Groups of animals in which 4TRP was administered directly into the ventricular system of the brain showed increased latency to the onset of myoclonic and generalized seizures as compared to groups of animals that received vehicle. These results corroborate those obtained in the behavioural experiments reported in this study. In general, mice receiving systemic 4TRP showed decreased seizure duration and significantly reduced seizure occurrence in comparison with the untreated group.

The ability to inhibit seizures in models of chemical induction by PTZ and picrotoxin suggests that 4TRP may interfere directly or indirectly with GABAergic neurotransmission, potentiating the action of gamma-aminobutyric acid (GABA).

GABA is a brain neurotransmitter that is derived from glutamate by the action of glutamic acid decarboxylase (GAD). Therefore, to better understand the involvement of the GABAergic system in the effects of 4TRP, we tested 4TRP's effects on seizures induced by the GAD inhibitor 3-mercapto-propionic acid (3-MP) [26]. Inhibition of GAD causes convulsions in animals by reducing the available GABA concentration, which directly alters inhibitory transmission mediated by GABA [26, 27].

The ability of a drug to inhibit or block 3-MP-induced seizures is a well-established indicator of the involvement of the GABAergic system in its effects.

The GABA_A_ receptor is a target for many anticonvulsant drugs [28]. Therefore, to evaluate the participation of the benzodiazepine site of the GABA_A_ receptor in the anticonvulsant effect of 4TRP, we tested this compound in the presence of flumazenil (FLU), which is a selective antagonist for the benzodiazepine site of the GABA_A_ receptor.

The effect of 4TRP was not altered by the presence of FLU. Therefore, although other modulatory sites existing on the GABA_A_ receptor could be involved, the anticonvulsant effect of 4TRP was not mediated by direct interaction with the GABA_A_ receptor benzodiazepine site. As expected, the anticonvulsant effect of diazepam was completely antagonized by pretreatment with FLU [29].

Figure 9(X) shows EEG recordings of the occurrence of clonic seizures, characterized by the appearance of multiple spikes, as well as slow waves typical of myoclonic seizures. The high amplitude (2-3 Hz) activity shown in this figure is a feature of generalized seizures, and thus the seizures elicited by PTZ in the behavioural tests were confirmed by physiological measurements [30].

The quantitative results presented in Figures 6, 7, and 8 are supported by the graphical representations shown in Figure 9, which illustrate that the 4TRP-treated groups showed less severe electrographical alterations. This reduction in the severity of symptoms in the recordings (fewer spikes) was associated with attenuation of the severity of behavioural seizures [30, 31]. These results indicate that 4TRP reduced the severity of paroxystic activity, but its effects were evaluated primarily with respect to generalized seizures.

The capacity of 4TRP to inhibit seizures induced by electrical stimulation [13] indicates that its mechanism of action may be related to the inhibition of neuronal firing through blockade of voltage-gated Na^+^ or Ca^2+^ ion channels.

The reduction in PTZ-induced seizure behaviour by 4TRP observed in EEG results suggests that the decrease in paroxysmal activity produced by 4TRP was mediated through its interference with GABAergic neurotransmission. However, more detailed pharmacological studies are needed to better elucidate this mechanism.

The rapid depolarisation of the neuronal membrane in a disorderly manner is widely present in convulsive disorders and is intimately related to the participation of voltage-activated sodium channels (Nav) [32]. Mutations in these channels are associated with the development of some types of epilepsy [33, 34].

4TRP inhibits voltage-gated Na^+^ current in dissociated dorsal root ganglion neurons. A large variety of Nav subtypes from DRG cells, including tetrodotoxin- (TTX-) sensitive channels Nav1.1, Nav1.2, Nav1.6, and Nav1.7 and TTX-resistant channels Nav1.8 and Nav1.9, have been evaluated for involvement in epilepsy [35, 36].

4TRP at a concentration of 1.0 mM significantly inhibited the Na^+^ current through the voltage-dependent sodium channel in vitro. This finding suggests that Na^+^ current inhibition is likely to be the major mechanism by which neuronal excitability and convulsive processes are impaired by 4TRP.

To demonstrate the reversibility of the effects of 4TRP, the exposure time was limited to 2.5 min. This short duration of exposure may have influenced the effective concentration, causing an apparent decrease in pharmacological potency for the 100 *μ*M concentration. Longer exposures could demonstrate better efficacy using lower concentration ranges. The compound eugenol, for example, takes about 12 min to inhibit voltage-gated Na^+^ channels [37].

Essential oils components, such as terpineol [38], estragole [39], 1,8-cineole [40], and linalool [22], have been reported to produce effects on the CNS [41] at similar concentration ranges and in similar experimental models to those used in this study. The essential oil of* Alpinia zerumbet*, of which 4TRP is a major component, alters electrophysiological parameters [42].

## 5. Conclusions

Through behavioral and electrophysiological models, it is concluded that 4TRP demonstrates a potential anticonvulsant effect, with ability to cause a significant protection against induced seizures. The action of this monoterpenoid is directly or indirectly related to the GABAergic system but does not act on the same binding site for benzodiazepines. Additionally, it is able to inhibit significant current voltage-dependent sodium channels, effects possibly related to changes in neuronal excitability as a consequence of modulation of these channels.

These results support the importance of the search for new epilepsy treatments in the vast array of available natural resources. Because of the wide range of bioactive phytochemicals and their diverse effects, this search is a challenging endeavour but one that has significant potential to discover new drugs.
